# Isolated Injury to Prepuce After Motor Vehicle Collision

**DOI:** 10.7759/cureus.8451

**Published:** 2020-06-05

**Authors:** Trina K Capelli, Anupam K Gupta, Monica I Burgos, Nir Hus

**Affiliations:** 1 General Surgery, Saint George's University, Boca Raton, USA; 2 Surgery, Charles E. Schmidt College of Medicine, Florida Atlantic University, Boca Raton, USA; 3 Internal Medicine, Universidad Autonoma de Guadalajara, Guadalajara, MEX; 4 Surgery, Delray Medical Center, Delray Beach, USA; 5 Surgery, Florida Atlantic University, Boca Raton, USA

**Keywords:** prepuce injury, penile injury, motor vehicle collison, blunt injury to genitalia, circumcision

## Abstract

A four-year-old male restrained passenger in the rear seat was in a motor vehicle collision. He had pain and swelling in the tip of the penis with the inability to micturate. The patient on evaluation had an isolated injury of the prepuce. The patient underwent an evaluation to rule out underlying penile/urethra and associated injury of kidney and bladder. The patient had an isolated injury to prepuce, causing urinary retention for which he underwent circumcision.

## Introduction

It is unusual to have an isolated injury of prepuce after a motor vehicle collision. Preputial injury is usually in association with other injuries [1]. Isolated injury to the prepuce is with zipper injuries commonly [2]. A motor vehicle collision and isolated preputial injury need an elaborate workup to rule out urethral/additional injuries. After a complete workup to rule out associated injuries, because of the isolated injury to prepuce, the damaged foreskin was debrided, and circumcision performed. This paper aims to show that with blunt injury mechanism, it is essential to rule out underlying injury, which could have a severe impact and is an unusual cause for isolated preputial injury.

## Case presentation

**Figure 1:**
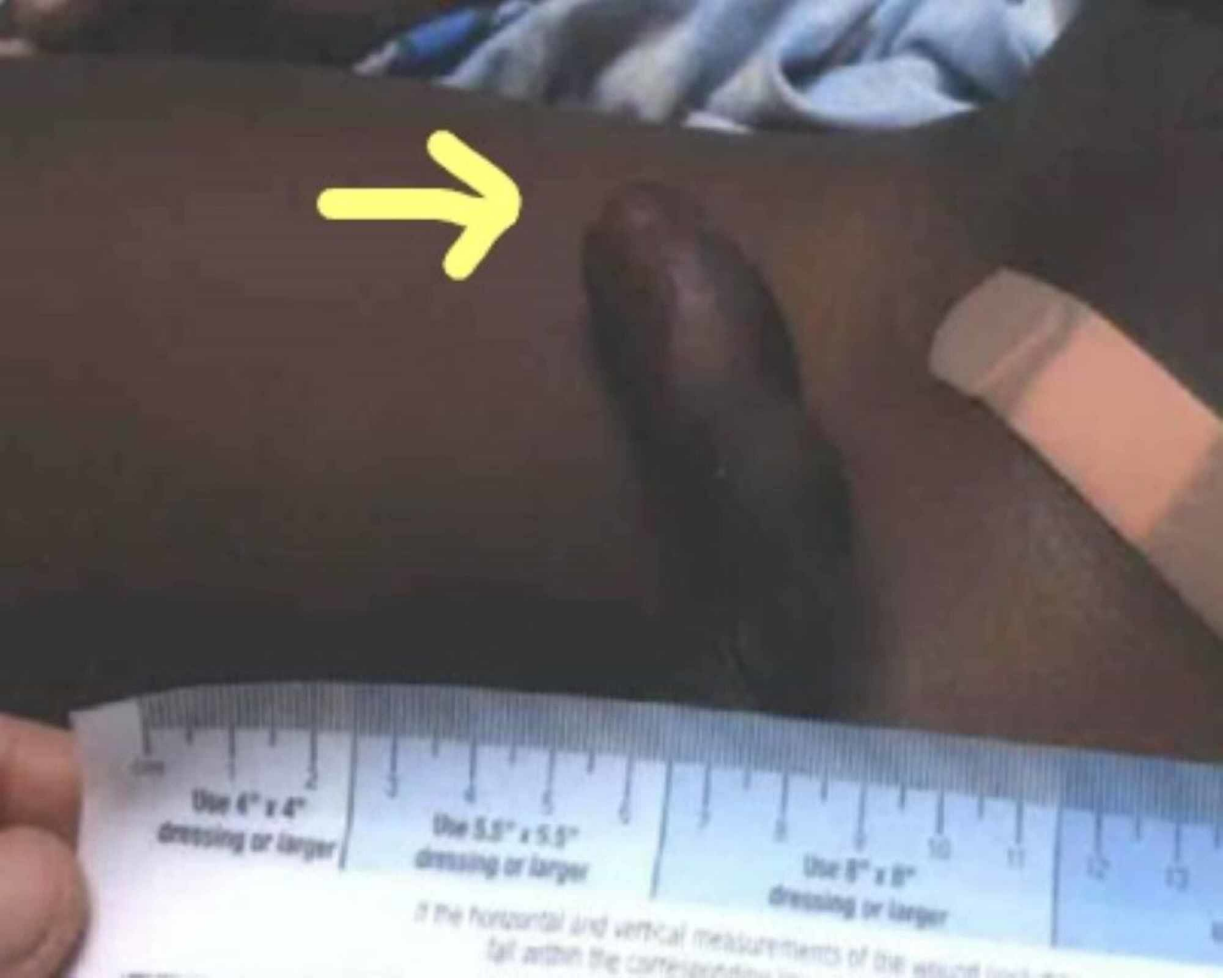
Swollen prepuce with urinary retention.

A four-year-old male boy was in the rear seat in a motor vehicle collision. He was in a car seat at the time of the accident. The patient got evaluated in the trauma emergency room at our hospital at Delray Medical Center in Florida. The patient had an unremarkable primary survey with stable vitals. In the secondary survey, the patient had a Glasgow Coma Scale of 15. No evidence of deformity and head-to-toe examination revealed mild erythema over the prepuce (Figure 1).

The child was unable to void and complained of pain. A pelvic x-ray showed no evidence of pelvic fracture, and CT of the abdomen and pelvis revealed no injury. In the operating room under general anesthesia, a hematoma with skin tears was visible on retracting the prepuce. The external urethral meatus showed no evidence of injury or congenital malformation. After confirming no damage, a retrograde urethral cystogram showed no evidence of injury to the urethra, and with the retrograde flow in the bladder. In view of paraphimosis with edema, causing urinary retention, the patient underwent circumcision to excise damaged foreskin with the placement of a Foley catheter. The postoperative course was uneventful, and the patient voided clear urine the next day. The patient got discharged home on postoperative day 2.

## Discussion

Motor vehicle accidents are one of the leading causes of death in the United States. Each year, motor vehicle accidents take the lives of more than 40,000 people in the United States, resulting in 2.7 million emergency department visits [3]. Motor vehicle-related injuries range from scrapes, cuts, head trauma, fractures, internal bleeding, and, less frequently, penile abrasions [4]. Preputial injuries are seen in association with other injuries. According to the National Electronic Injury Surveillance System (NEISS), genital injuries represented 0.6% of all pediatric injuries [5].

Preputial injury is commonly iatrogenic in origin and follows procedures such as circumcision and hypospadias surgery [1]. Non-iatrogenic causes include most commonly being zipper injuries, child abuse, self-mutilation, animal attack, religions, congenital cause, and car accidents [2-14]. Zipper injuries are the only consistently reported etiology in the pediatric population [2]. Hair tourniquet can cause injury to prepuce, but the majority of hair tourniquet injuries involve the penile shaft [5,6]. Genital self‐mutilation (GSM), also known as Klingsor syndrome (self-mutilation by a psychiatric patient with religious delusions), is a rare phenomenon, with little over a hundred cases identified in the literature [6]. Self‐inflicted wounds range from simple lacerations of the external genitalia to complete amputation of the penis and testes, presenting a significant challenge for physicians in their management [7].

The prepuce is a sleeve of skin that covers the end of the penis in its flaccid state; penile erection retracts prepuce to expose the glans [8]. Its function is to protect the sensitive tip of the flaccid penis [9]. The foreskin is at least a third of the penile skin. It protects the glans from abrasion and contact with clothes. The foreskin maximizes sexual pleasure by sliding up and down on the shaft, thereby stimulating the glans [10]. The preputial skin is remarkable for its thinness and its mobility. These factors account for the vulnerability of the preputial skin to injury [11]. The elasticity of genital skin means it is usually possible to manage the loss of a moderate amount of penile skin; because of this elastic tissue from the prepuce, it has utility in the reconstruction of hypospadias [12].

There are no specific algorithms for treating severe penile trauma, and no particular algorithm is appropriate for all types of penile injury [7]. Consideration should be given to the type of injury, severity, site, and patient history [7]. The main reasons for this include the diverse nature of injury mechanism and various anatomical landmarks [13]. The American Association for the Surgery of Trauma (AAST) has guidelines for penile injury (Table 1) [13].

**Table 1:** American Association for the Surgery of Trauma (AAST). Organ injury scale of penile injury. *Advance one grade for multiple injuries up to grade III. Advance one grade for bilateral lesions up to grade V.

Grade*	Description of injury
I	Cutaneous laceration/contusion
II	Buck’s fascia (cavernosum) laceration without tissue loss
III	Cutaneous avulsion/laceration through glans/meatus/cavernosal or urethral defect <2 cm
IV	Cavernosal or urethral defect >2 cm/partial penectomy
V	Total penectomy

Using the AAST injury scale (Table I), our patient had a type I injury. Our patient had mild erythema over the prepuce with no evidence of deformity or evidence of pelvic fracture or involvement of the corpora. Because of paraphimosis and urinary retention, the patient underwent circumcision that would allow him to have a better cosmetic outcome.

## Conclusions

Isolated preputial injury after a motor vehicle collision causing urinary retention is unusual. One should rule out the underlying injury to urethra before performing circumcision.
